# Crosstalk Between Dermal Fibroblasts and Dendritic Cells During Dengue Virus Infection

**DOI:** 10.3389/fimmu.2020.538240

**Published:** 2020-10-23

**Authors:** Alfredo E. Montes-Gómez, Julio García-Cordero, Edith Marcial-Juárez, Gaurav Shrivastava, Giovani Visoso-Carvajal, Francisco J. Juárez-Delgado, Leopoldo Flores-Romo, Ma. Carmen Sanchez-Torres, Leopoldo Santos-Argumedo, José Bustos-Arriaga, Leticia Cedillo-Barrón

**Affiliations:** ^1^ Departamento de Biomedicina Molecular Centro de Investigación y de Estudios Avanzados, del Instituto Politécnico Nacional, Ciudad de México, México; ^2^ Departamento de Biología Celular Centro de Investigación y de Estudios Avanzados, del Instituto Politécnico Nacional, Ciudad de México, México; ^3^ Departamento de Cirugía, Hospital Santa María Ticomán, Ciudad de México, México; ^4^ Unidad de Biomedicina, Laboratorio de Biología Molecular e Inmunología de arbovirus, Facultad de Estudios Superiores-Iztacala, Universidad Nacional Autónoma de México, Tlalnepantla, México

**Keywords:** antiviral microenvironment, dendritic cells, dengue virus, dermal fibroblasts, monocytes, type I interferons

## Abstract

Dengue virus infection (DENV-2) is transmitted by infected mosquitoes *via* the skin, where many dermal and epidermal cells are potentially susceptible to infection. Most of the cells in an area of infection will establish an antiviral microenvironment to control viral replication. Although cumulative studies report permissive DENV-2 infection in dendritic cells, keratinocytes, and fibroblasts, among other cells also infected, little information is available regarding cell-to-cell crosstalk and the effect of this on the outcome of the infection. Therefore, our study focused on understanding the contribution of fibroblast and dendritic cell crosstalk to the control or promotion of dengue. Our results suggest that dendritic cells promote an antiviral state over fibroblasts by enhancing the production of type I interferon, but not proinflammatory cytokines. Infected and non-infected fibroblasts promoted partial dendritic cell maturation, and the fibroblast-matured cells were less permissive to infection and showed enhanced type I interferon production. We also observed that the soluble mediators produced by non-infected or Poly (I:C) transfected fibroblasts induced allogenic T cell proliferation, but mediators produced by DENV-2 infected fibroblasts inhibited this phenomenon. Additionally, the effects of fibroblast soluble mediators on CD14^+^ monocytes were analyzed to assess whether they affected the differentiation of monocyte derived dendritic cells (moDC). Our data showed that mediators produced by infected fibroblasts induced variable levels of monocyte differentiation into dendritic cells, even in the presence of recombinant GM-CSF and IL-4. Cells with dendritic cell-like morphology appeared in the culture; however, flow cytometry analysis showed that the mediators did not fully downregulate CD14 nor did they upregulate CD1a. Our data revealed that fibroblast-dendritic cell crosstalk promoted an antiviral response mediated manly by type I interferons over fibroblasts. Furthermore, the maturation of dendritic cells and T cell proliferation were promoted, which was inhibited by DENV-2-induced mediators. Together, our results suggest that activation of the adaptive immune response is influenced by the crosstalk of skin resident cells and the intensity of innate immune responses established in the microenvironment of the infected skin.

## Introduction

Dengue virus (DENV-2) is a highly prevalent vector-borne pathogen present in tropical and subtropical regions. It belongs to the genus *Flavivirus* and has a positive-sense single-stranded RNA genome with an open reading frame that encodes seven non-structural proteins and three structural proteins (1). DENV-2 is transmitted through the bite of the female *Aedes aegypti* mosquito during the feeding process in the skin (2). Thus, resident skin cells are the first target of infection and actively participate in the immune response and wound healing process (3). Our group and others have demonstrated that keratinocytes (4, 5), fibroblasts (6, 7), Langerhans cells (LC) (8), dermal dendritic cells (9), and endothelial cells (10) are permissive to different arboviral infections. Dendritic cells (DCs) are one of the most studied cell types as a primary target for dengue. Upregulated expression of MHC-I, MHC-II, CD80, CD86, CD83, and CD40 has been observed in both DENV infected and bystander DCs (11). Furthermore, Langerhans cells and dermal DCs infected with DENV-2 trigger the secretion of proinflammatory cytokines such as IL-1β, IL-6, and TNF-α as well as initiate a strong interferon-α (IFN-α) response (12, 13). Regarding other myeloid cells, recent work with DENV-2 infected mice has shown that monocytes expressing CCR2 are recruited to the dermis and differentiate into dendritic cells, which suggests that these events contribute to the pathology of the disease, since more target cells are available for the virus to infect (14).

Studies with human dermal fibroblasts (HDF) showed that infection with either DENV-2 or Zika virus (ZIKV) triggered the synthesis of antimicrobial peptides and IFN-β; this induces an antiviral state through the expression of molecules such as MX1, ISG15, and OAS2 (7, 15). Fibroblasts have multiple functions depending on their location site; thus, fibroblasts isolated from the synovium, skin, bone marrow, and lymph nodes show variable differentiation and proliferation capacities. Hence, fibroblasts are capable of producing numerous immune modulators, such as peptide growth factors, cytokines, and chemokines (16). Interestingly, data from different groups using noninfectious models have shown that dermal fibroblasts can shape T cell responses (17).

Previous work has shown that the microenvironment of stromal cells, such as fibroblasts, directly modulates the duration of inflammatory responses in rheumatoid arthritis (18). Furthermore, it was recently described that DENV-2 infection is controlled in cocultures of human dermal fibroblasts and human dermal microvasculature endothelial cells (HDMEC). The production of soluble mediators such as IFN-β, RANTES, and IL-6 is enhanced only in the HDF : HDMEC coculture conditions. Such mediators also affect the activation phenotype of endothelial cells: HDMECs cocultured with DENV-2 infected fibroblasts express an activated phenotype and allow leukocyte transmigration (19). These data provide insight into how two types of skin cells communicate with each other to initiate an antiviral response in one cell type and promote activation in another for later recruitment of cells to the site of infection. In 2018 Duangkhae et al. provided valuable information regarding the complex skin microenvironment during DENV-2 inoculation in human skin explants. They found that the epidermal compartment is primarily infected as early as 6 hours, and by 12 hours the infection reached the dermis; Langerhans cells, keratinocytes, fibroblasts, the endothelium, macrophages, and mast cells are infected, as observed using tissue immunofluorescence assays (20). However, not only an antiviral immune response is observed; proinflammatory cytokines are also highly expressed in the infected skin. IL-1β produced in the epidermis enhances infection and recruitment of myeloid cells to the site of infection, while neutralizing antibodies against IL-1β show a reduction in the infection on LC, dermal DCs, and macrophages (20). Although some work has attempted to understand the immune response in the skin during dengue infection, the influence of the mediators produced by the different cell types that reside in the skin is not fully elucidated. Understanding the interaction and further communication between different cell types could give us better insight into how infection is controlled or promoted at early stages before the virus spreads from the skin throughout the body.

In this study, we evaluated the role of the crosstalk between HDF and monocyte derived DCs (moDC) in the control of DENV-2 infection using HDF:moDC autologous cocultures and HDF conditioned media (FCM). We observed that moDCs promote the control of infection in HDF mainly by enhancing the production of type I interferons; at the same time, infected or activated fibroblasts induce variable levels of maturation of dendritic cells (partially matured DC [pmDC]). Further, we found that pmDC incubated with conditioned media from uninfected and polyinosinic-polycytidylic acid (Poly [I:C]) stimulated fibroblasts can promote T cell proliferation; however, pmDCs by infected fibroblasts completely hampered T cell proliferation. Such cells are thought to act as secondary targets for DENV-2 after initial replication in resident skin cells.

Our results suggest that the interaction between HDF and DC during the first stages of infection with DENV could shape the quality of activation of T cell response, and this early interaction seems to be influenced by the antiviral and pro-inflammatory response in the skin. However, antigen presentation by DC infected in the skin could be tampered by some strains of DENV. Additionally the information provided by our work helps to understand the initial events during DENV infection and might contribute to the understanding of why some infected patients develop only a mild disease, while others are at higher risk to develop severe disease.

## Materials and Methods

### Ethic Statement

Seven healthy donors (two male and five females) were enrolled for this study with an age range between 25 and 55 years. Human skin biopsies and PBMC samples from the donors were treated anonymously with the approval of the ethics committee. Informed consent was received and signed from participants before taking the samples. All data were collated and analyzed anonymously, and no identifiable information was collected.

### Virus and Cells

New Guinea reference strain was propagated in C3/36 cells, derived from *Aedes albopictus*, and grown in MEM media pH 7.2, supplemented with 10% fetal bovine serum (FBS), sodium pyruvate, L-Glutamine, non-essential aminoacids (NEAA), and incubated at 34°C. All cell culture materials were obtained from Gibco (Carlsbad, CA).

DENV2 stock was prepared by infecting an 80–85% confluent monolayer of C6/36 cells during 24–48 hours until the cytopathic effect was observed. Then the supernatant was collected and cleared of cells by centrifugation at 2000 rpm during 10 minutes at 4°C and concentrated using 0.22 μm Amicon columns to 1/10 from its starting volume. For cryoprotection, 1/10 of SPG stabilizer (2.18 mM sucrose, 38 mM monobasic K_2_HPO_4_, 72 mM dibasic K_2_HPO_4_, 60 mM L-glutamic acid) was added, then aliquoted and stored at -80°C until further use.

Vero cells and dermal fibroblasts were cultured in RPMI media pH 7.8 (Gibco, Carlsbad, CA) supplemented with 5% FBS, L-Glutamine, NEAA, vitamins, and incubated in a CO_2_ atmosphere at 37°C.

### Virus Titration

The virus was titrated by plaque forming assay technique using Vero cells. Briefly, 10-fold serial dilutions of virus stock or cell supernatants in Hank’s salt solution (Gibco, Carlsbad, CA) were used to infect confluent monolayers of Vero cells in 24-well plates. After incubation at 37°C for 1 h, the infected cells were overlaid with RPMI with 1% methyl cellulose (Sigma-Aldrich), 2% FBS, and 2 mM L-glutamine (Gibco, Carlsbad, CA). After 5 days, the monolayers were washed with phosphate buffered saline (PBS), then fixed and permeabilized with 80% ice-cold methanol for 15 minutes, then blocked with 5% low fat powdered milk diluted in PBS; the virus was identified with a mouse anti pan-flavivirus 4G2 antibody and with an anti-mouse-peroxidase secondary antibody. After the secondary antibody incubation, plaques were developed with peroxidase substrate (TrueBlue, Seracare, Milford, MA, USA). Plaques were counted manually and titers are expressed in PFUs/mL.

### Study Subjects

Human skin biopsies and PBMC samples from 7 healthy donors were enrolled for this study. The samples were treated anonymously with the approval of the ethics committee. Informed consent was received and signed from participants before obtaining the samples. All data were collated and analyzed anonymously, and no identifiable information was collected.

### Isolation of Dermal Primary Fibroblasts

Human dermal fibroblasts (HDF) were isolated from the skin biopsies from the volunteers; a 3 mm explant was excised from the forearm or back and washed with PBS containing penicillin (400 mg/mL) and streptomycin (400 U/mL) to remove all blood remnants and incubated overnight at 4°C in dispase (1 mg/mL, Sigma Aldrich, St. Louis, MO) with the epidermis facing down in a 24-well plate. The next day, the epidermis was removed with clamps and the dermis was digested in a cocktail of collagenases (0.5 U/mL Cat. C8176) and dispase (4 U/mL Cat. D4693) for 3 hours with strong agitation (both enzymes were obtained from Sigma Aldrich). The dermis was then washed with PBS and digested with a solution of 0.05% EDTA and trypsin once more for 30 minutes with agitation. Finally, the digested tissue was cultured on a 6-well plate in fibroblast medium with epidermal growth factor (10 ng/mL) for 5–10 days until a monolayer was observed. The cells were then passed and a fibroblast stock of each donor was stored in liquid nitrogen for further experiments. Additionally, surface marker expression was checked on the primary cell cultures with the antibody 1B10 (Cat. F4771 Sigma Aldrich).

### *In Vitro* Generation of Dendritic Cells From Blood Monocytes

Monocyte derived dendritic cells (moDC) were differentiated from CD14^+^ peripheral blood monocytes obtained from the same skin donors. Briefly, 20 mL of total blood were obtained from each donor, and PBMCs were isolated by density gradient centrifugation using Ficoll-Hypaque (Histopaque 1077, Sigma Aldrich Chemical Co., St. Louis, MO); CD14+ monocytes were positively selected from PBMCs using anti-CD14 microbeads (Cat. 130-050-201) (Miltenyi Biotech, Auburn, CA) and a magnetic cell separator (MACS) (Miltenyi Biotech, Auburn, CA) according to manufacturer’s instructions. Monocytes from different donors were seeded in plastic flasks at a density of 1x10^6^ per ml of differentiation monocyte culture media (RPMI supplemented with 10% FBS, sodium pyruvate, L-Glutamine, NEAA, vitamins, recombinant human GM-CSF ([50ng/mL, Peprotech, London, United Kingdom] and recombinant IL-4 [30 ng/mL, Peprotech, London, United Kingdom]) in a CO_2_ atmosphere at 37°C. Cytokines were replenished every two days and cells were used on day 6 as immature DC. The appropriate phenotype of immature DC was confirmed by flow cytometry prior to each experiment (all DCs were CD14^-^, CD1a^+^, HLA-DR^+^, CD83^-^, CD86^+^).

### Isolation of T Cells From Peripheral Blood

The CD14^-^ fraction obtained from the monocyte isolation was used for a negative selection of T cells using a Pan T Cell Isolation Kit (Cat. 130-091-156, Miltenyi Biotech, Auburn, CA) and a magnetic cell separator (MACS; Miltenyi Biotech, Auburn, CA) according to the manufacturer’s instructions.

### Dengue Infection of the Cell Cultures

For co-cultures and infection of HDFs and moDC were obtained from the same donor and seeded together for co-culture experiments in a 5:1 ratio in 24-well plates and incubated for 12–18 h prior to infection with DENV-2. The cells were infected with DENV-2 at a multiplicity of infection (MOI) of 5 with DENV-2 by removing the culture media and adding the virus diluted in fresh media in a final volume of 200 μL per well for two hours with rocking every 15 minutes. The inoculum was then removed and 500 μL of fresh media was added. All infections were incubated for 24 or 48 h unless otherwise specified.

### Transfection of Dermal Fibroblasts

For *in vitro* activation of HDFs were seeded in a 6-well plate at a confluence of 240,000 cells per well and transfected with 10 μg of the ssRNA analog Poly(I:C) mixed with Lipofectamine 2000 reagent (Invitrogen Life Technologies) in a final volume of 800 μl of serum free media for four hours at 37°C. The cells were then washed with PBS 1X and cultured in complete media for 24 hours after transfection.

### Flow Cytometry

The dendritic cell phenotype was determined by flow cytometry using cell surface labelling. Briefly, DCs suspensions were blocked with an irrelevant immunoglobulin to reduce non-specific binding; cell membrane labelling to detect differentiation and maturation markers was performed using and-hCD14-PE and anti-hHLA-DR-FITC, both from R&D (Minneapolis, MN); anti-hCD1a-APC, anti-hCD83-PE, and anti-hCD86-APC were obtained from BD Pharmigen (San Jose, CA). To detect dengue infected cells, we used the 3H5 antibody, kindly donated by Dr. Stephen Whitehead, that binds domain III of the envelope proteins of dengue serotype 2. The following isotype controls were included in each experiment: APC-mouse IgG isotype control, FITC-mouse IgG isotype control, and PE-mouse IgM isotype control, all from BD Pharmingen (San Jose, CA). For the analysis of cell suspensions, mono-cultures, and co-cultures, adherent cells were dethatched with trypsin-EDTA; cell suspensions were then fixed and permeabilized with Cytofix-Cytoperm solution (BD 554714) according to the manufacturer’s instructions. The mouse anti-DENV-2 3H5 primary antibody was added, followed by a fluorescently labelled secondary antibody Cy3 goat anti-mouse IgG (Life Technologies, Eugene, OR, USA). All incubations were made in ice. After a step of washing, cell suspensions were resuspended in PBA solution (PBS, 0.02% BSA, and 0.01% sodium azide) and acquired in a BD LSR II Fortessa flow cytometer (Becton Dickinson, Franklin Lakes, NJ. All data were analyzed using FlowJo 10 (Tree Star, Inc.). The results were statistically analyzed with Graph Pad Prism 6 software (CA, USA).

### Detection of Pro-Inflammatory Cytokines and Interferons

The supernatants from infected cells were collected at different times post infection and then interleukin-1β (IL-1β), IL-6, IL-8 and tumor necrosis factor-α (TNF-α) were determined by ELISA using the BD OptElA Human IL-1β ELISA Set (Cat. 557953), BD OptElA Human IL-6 ELISA Set (Cat. 555220), BD OptElA Human IL-8 ELISA Set (Cat. 555244. BD), and BD OptElA Human TNF-α ELISA Set (Cat. 555212), all obtained from DC Biosciences (CA, USA). Briefly, the plates were coated overnight with the capture antibody for each cytokine using carbonate buffer pH 9.5 at 4°C according to the suggested dilutions by the manufacturer. The plates were then blocked for one hour with PBS-10% FBS. The standards and the supernatants were diluted in blocking buffer and added to the wells for 2 hours at room temperature. The detection antibodies and Streptavidin-peroxidase (SAv-HRP) conjugates were added together, diluted in blocking buffer, and incubated for one hour at room temperature. OPD substrate solution was added to each well and incubated at 37°C for 15 minutes, then the reaction was stopped with H₂SO₄ 2N. IFN-β was quantified using the ELISA kit LumiKine hIFN-β (Cat. luex-hifnbv2. Invivo Gen. San Diego, USA). The protocol followed was similar to inflammatory cytokines, except that blocking was performed for 2 hours at 37°C with 2% BSA. The standard or supernatants were diluted in 1% BSA and incubated for 2 hours at 37°C followed by the Lucia conjugated detection antibody. The plates were developed with the QUANTI-Luc™ assay solution and read immediately in a chemiluminescence plate reader (Infinite F500 TECAN).

### CFSE-Dilution Assay

T lymphocytes were labelled with 15 μM CFSE for 30 minutes, and then T cells were washed twice with PBS 10% FBS. Labeled T cells were co-cultured at a 10:1 ratio (T: moDC) in the presence of moDC previously treated with FCM, or those activated with TNF-α/LPS. T cells alone were stimulated with PMA/ionomycin and used as positive controls. Unstimulated T cells were used as negative controls.

## Results

**Figure 1:**
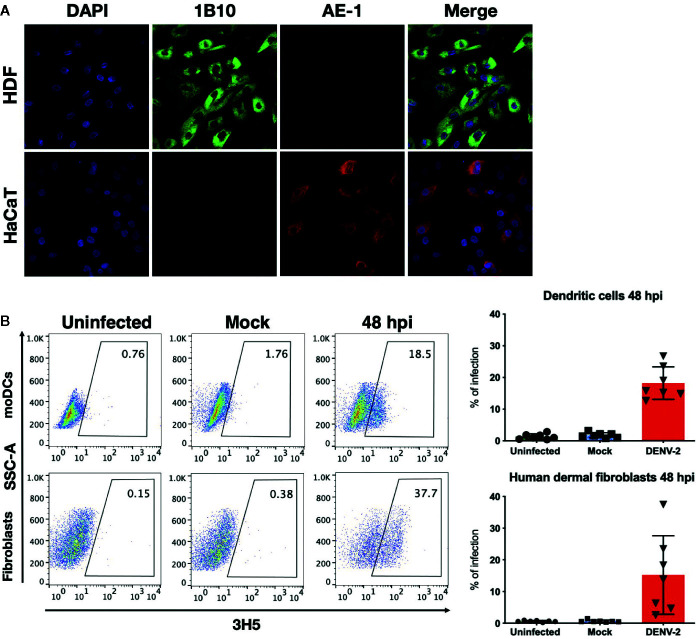
Characterization of HDF and permissiveness to DENV infection in primary HDF and moDC. **(A)** Immunofluorescence staining of HDF and HaCaT cells with 1B10 (green) and AE-1 (red) antibodies that label fibroblasts and keratinocytes, respectively. **(B)** Confluent monolayers of fibroblasts or moDCs were infected at a multiplicity of 5 and 2, respectively, for 48 hours. Subsequently, flow cytometry staining was performed to determine the percentage of infected cells based on the expression of the Envelope viral antigen. Error bars indicate the standard deviations for the three independent experiments of each donor, n = 7.

First, the dermal fibroblast model was characterized by assessing the expression of fibroblast antigen with the antibody 1B10 and the lack of epithelial marker expression, such as cytokeratins (antibody AE-1, also known as Pan-cytokeratin). As shown in **Figure 1A**, dermal fibroblasts expressed the antigen recognized with the antibody 1B10 and cytokeratins were not detected. In this work, primary skin fibroblast cultures and moDC were established and subsequently infected with DENV-2 at a multiplicity of infection (MOI) of 5 and 2, respectively. The presence of the viral E protein was evaluated in those cells using flow cytometry and the DENV infectivity rate of these cells was observed to be between 9% and 50% at 48 hpi in all of the analyzed primary cultures from different donors (**Figure 1B**).

### Dermal Fibroblasts Infected With DENV-2 Promote the Partial Maturation of Dendritic Cells and Resistance to Infection

**Figure 2:**
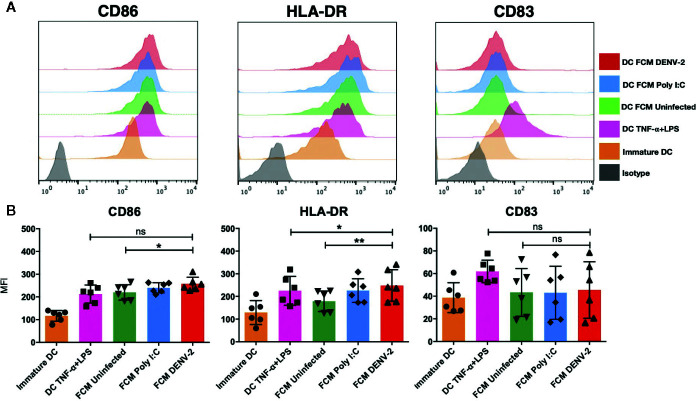
Fibroblasts’ conditioned media promote the partial maturation of moDC. Conditioned media of uninfected (UI), Poly I:C transfected (PIC), or DENV-2 infected fibroblasts was used to treat immature moDC for 24 hours. Maturation markers were evaluated using flow cytometry. Controls included moDC cultured without stimulus and stimulated with TNF-α+LPS. **(A)** Representative histograms of the maturation markers CD86, CD83, and HLA-DR. **(B)** Mean fluorescence intensity (MFI) of the maturation markers evaluated for all six donors. Error bars indicate the standard deviations for the three independent experiments of each donor; n = 6. ns, no significant. *P < 0.05 and **P < 0.01.

**Figure 3:**
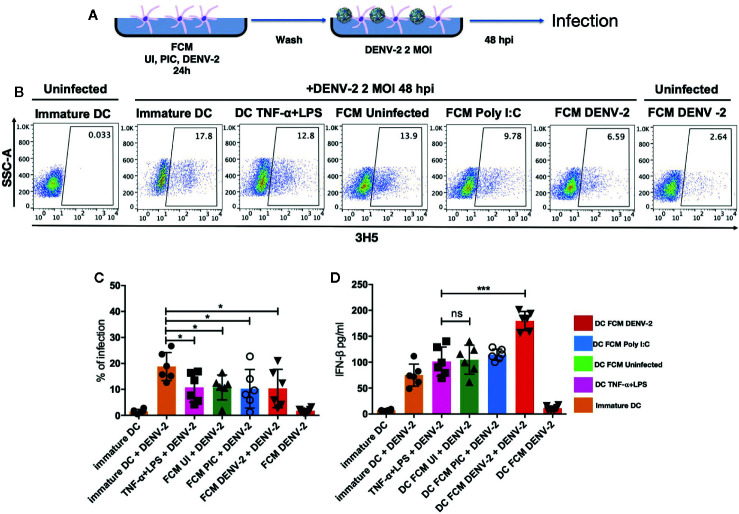
DENV-2 infection is inhibited in fibroblast-matured moDC. moDCs treated with all three FCM for 24 hours were washed and infected with DENV-2 at 2 MOI for 48 hours. The infection rate was assessed with intracellular staining using flow cytometry. **(A)** Schematic representation of the maturation and infection of moDCs. **(B)** Representative dot plot from one donor where DENV-2 infection rate was observed. **(C)** Percentage of infection among all donors as measured using flow cytometry, which assessed the expression of the viral envelope protein. **(D)** IFN-β secretion by fibroblast-matured moDC that were infected with DENV-2, as measured using ELISA. Error bars indicate the standard deviations for the three independent experiments of each donor; n = 6. ns, no significant. *P < 0.05 and ***P < 0.001.

Dendritic cells have been shown to be the main link between the innate and the adaptive immune response (21); skin DCs are also sensitive to microenvironment modifications during any pathological or infectious process. It has been demonstrated that dermal fibroblasts physically interact with DCs in atopic skin lesions, promoting maturation of DCs (mDC), as observed by the upregulation of antigen presentation machinery such as CD80, CD83, CD86, and HLA-DR (17). Therefore, we analyzed the effect of fibroblast conditioned media (FCM) from a total of seven healthy donors treated under different conditions (uninfected HDF [FCM-UI], HDF transfected with Poly [I:C] [FCM-PIC], or DENV-2 infected HDF [FCM-DENV-2]). Immature moDC were cultured in the above described FCM and control moDC were treated with TNF-α/LPS for 24 hours, followed by flow cytometry analysis of CD86, CD83, and HLA-DR expression (**Figure 2A**). Our data showed that in all donor cultures, the expression of CD86 and HLA-DR was upregulated; however, in the cells treated with FCM-DENV-2 the expression of these molecules was observed in some cases at levels similar to moDC treated with TNF-α/LPS. This suggested that the mediators produced by dengue infected fibroblasts induced variable levels of DC maturation. Both CD86 and HLA-DR are important for antigen presentation, as well as CD83, which did not show a statistically significant difference among the different conditioned media compared with the maturation control. However, three of the FCM-DENV samples induced the upregulation of CD83 (**Figure 2B**). Next, we evaluated whether the mediators produced by infected fibroblasts would render matured moDCs more resistant to DENV-2 infection, given that DENV-2 induces a strong IFN-β response in HDF. Briefly, moDCs were incubated with FCM for 24 hours to induce moDC maturation; the cells were then washed and infected with DENV-2 at 2 MOI for 48 hours. The infection was assessed using flow cytometry based on the expression of the E protein. **Figure 3A** shows the flow chart of DENV infected moDCs followed for maturation. A representative dot plot in **Figure 3B** indicates that moDC matured by FCM-DENV-2 were able to control DENV-2 infection just as efficiently as the moDC matured with TNF-α+LPS (**Figure 3C**), probably due to the HDF production of type I interferons as previously reported by our group (7). Furthermore, the secretion of IFN-β by the infected moDCs was also assessed; FCM-DENV-2 not only promoted the control of DENV-2 infection but also promoted the type I interferon production by dendritic cells, as observed in **Figure 3D**. These data suggest that the microenvironment induced by DENV-2 infected fibroblasts promotes the control of DENV-2 infection and maturation of moDCs.

### Effect of moDC Matured With FCM on the Induction of Autologous T Cell Proliferation

**Figure 4:**
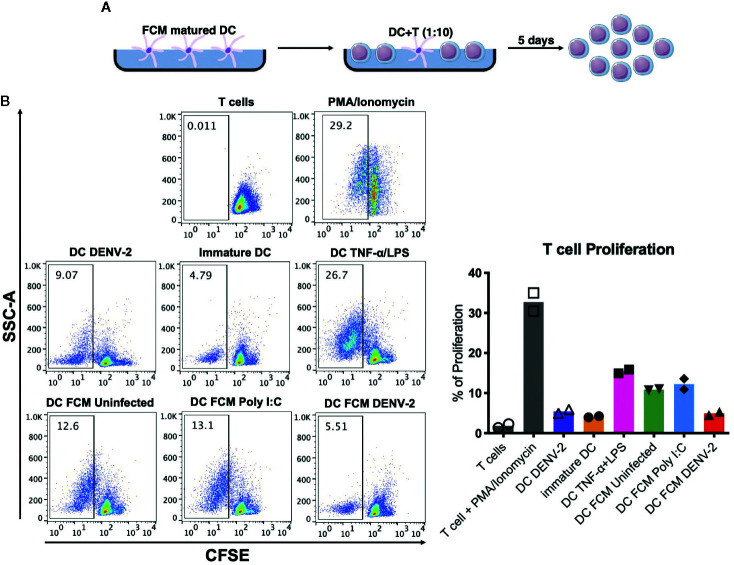
Dendritic cells matured by fibroblast soluble mediators fail to induce T cell proliferation. **(A)** Schematic representation of experimental conditions. **(B)** Representative dot plot of a CFSE assay where T cell proliferation was observed. Analysis of the T cell proliferation percentage by fibroblast matured DC for two different donors of each donor. ns, no significant.

To evaluate whether FCM (**Figure 4A**) matured DCs were able to induce the proliferation of autologous T cells, mature DCs were cocultured with autologous CFSE-labeled T cells. **Figure 4B** shows a representative dot plot of the T cells and the loss of CFSE fluorescence intensity as a result of T cell division in coculture with dendritic cells. **Figure 4B** shows the proliferation percentage observed in each coculture condition. Matured mDCs in the presence of FCM-UI and FCM-PIC induced T cell proliferation of around 12%, most likely in response to self-antigens; however, FCM-DENV-2 treated DCs were unable to induce this phenomenon. This is similar to immature DCs when they were infected directly with DENV-2. This suggests that in fibroblasts, DENV-2 promotes the secretion of mediators that inhibit the ability of dendritic cells to induce T cell proliferation. These data showed for the first time the effect of DENV-2 infected mesenchymal cells on the functionality of dendritic cells and their capability to inhibit a T cell response.

Even though the activation of autologous T cells by FCM matured DC was modest, this could suggest the evasion capability of the DENV strain used in our study to compromise antigen presentation in the DCs. This has been observed in yellow fever virus infection of DCs from humans and rhesus macaques, where DCs infected with the attenuated vaccine strain 17D stimulated CD4^+^T cells and the DCs infected with the wild type virus did not stimulate CD4^+^ T cells (22).

### Soluble Mediators Produced by DENV-2 Infected Fibroblasts Interfere With Monocyte Differentiation Into Dendritic Cells

**Figure 5:**
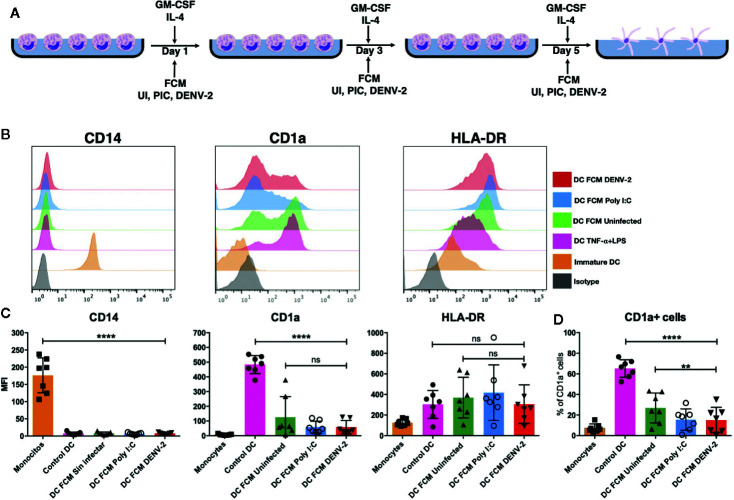
Fibroblasts suppress monocyte differentiation into dendritic cells. Fibroblast conditioned media was used to treat monocytes for 6 days in the presence of GM-CSF and IL-4, then CD14, CD1a, and HLA-DR expression levels were evaluated using flow cytometry. **(A)** Schematic representation of experimental conditions. **(B)** Representative histograms of moDCs from one donor showing the expression of CD14, CD1a, and HLA-DR under the treatments previously mentioned. **(C)** Graphic representation of MFI of cells expressing CD14,CD1a and HLA-DR on the moDC after differentiation. **(D)** Analysis of the percent of cells expressing CD1a on moDC. Error bars indicate the standard deviations for the three independent experiments of each donor; n = 7. ns, no significant. **P < 0.01 and ***P < 0.0001.

Given that monocytes are recruited into sites of inflammation to differentiate into new DCs (14), the role of fibroblast-produced mediators on autologous monocytes under differentiation into DCs was evaluated (**Figure 5A**). Conditioned media from fibroblasts, as mentioned before, were used to treat autologous monocytes and additionally they were stimulated with GM-CSF and IL-4 on days 1, 3, and 5. Differentiation into moDC was then evaluated and compared with control moDC (exposed only to differentiating cytokines). **Figure 5B** shows representative histograms of cells. **Figure 5C** shows that CD14 expression was almost completely downregulated, HLA-DR was upregulated, and CD1a, a moDC marker, was highly expressed in control moDCs. Meanwhile, all FCM in the presence of the differentiating cytokines caused a reduction in the expression of CD14, whereas CD1a did not reach the expression levels compared to control moDC. The expression of HLA-DR was upregulated in most donors as in control moDC (without FCM). When analyzing the percentage of CD1a^+^ cells, a reduction in this population was observed compared to control moDC (**Figure 5D**). Monocytes were also treated with all three FCM only to evaluate if fibroblast mediators were able to induce differentiation into moDC; however, little to no cell survival was observed by day 3 and this condition was excluded in further experiments (data not shown).

### DENV-2 Infection Rate Decreases in HDF:moDC Cocultures

**Figure 6:**
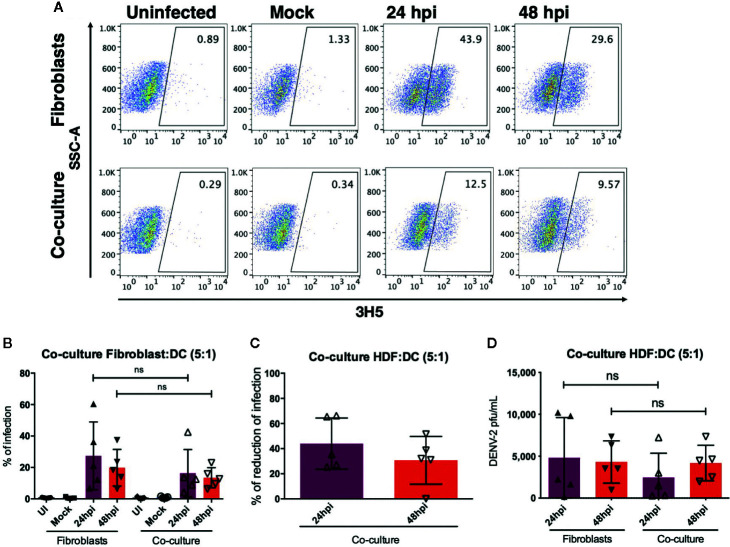
Dendritic cells promote control of DENV-2 infection by fibroblasts. Dermal fibroblasts alone or in coculture with autologous moDC in a 5:1 ratio were infected with DENV-2 for 24 and 48 hours, and then infection was evaluated based on the expression of the envelope protein using flow cytometry. **(A)** Representative dot plot of one donor showing the infection rate in monoculture and coculture. **(B)** Percent of DENV infected cells in the coculture and monoculture conditions. **(C)** Reduction of the infection based on the maximum infection rate of the monoculture infections. **(D)** Analysis of plaque forming units in the supernatants of infected cultures. Error bars indicate the standard deviations for the three independent experiments of each donor; n = 5. ns, no significant.

As mentioned previously, DENV-2 and other flaviviruses have been shown to infect resident skin and migratory cells *in vitro* and *ex vivo (* 7, 15, 20). Given the fact that each cell type in the skin produces a specific cytokine profile after sensing pathogens, the microenvironment will be greatly modified, even if a particular cell is not permissive to infection. To assess the microenvironment induced by dermal fibroblasts and dendritic cells, coculture experiments were conducted, and cytokine profiles and type I interferons were evaluated in every condition. First, HDF monocultures and autologous cocultures of HDF and dendritic cells (5:1 ratio) were infected with DENV-2 at 5 MOI for 24 and 48 hours and the percent of infection was evaluated using flow cytometry. **Figure 6A** shows a representative dot plot of uninfected cells, cells infected with an UV-irradiated DENV-2 (Mock), and DENV-2 infected monocultures and cocultures; the cells were stained with the 3H5 antibody that binds to the E viral protein. **Figure 6B** shows the wide variety of permissiveness to DENV infection in all five donors: at 24 hpi infection was barely detected in some donors, and it increased at 48 hours compared to HDF monocultures, where infection reached up to 60% of E positive cells. **Table 1** shows the wide permissiveness to infection of all HDF from different donors, both at 24 and 48 hpi. With this in mind, we determined the maximum rate of infection (%MRI) for each donor, and based on this, we calculated the percent reduction of infection (%RI) in the cocultures at both times. **Table 1** and **Figure 6C** show the reduction of infection in the cocultures; the samples from five donors showed a reduction in the infection rate of the total population up to 65% in the coculture conditions. Infectious viral particles were also quantified from the supernatants of the cocultures and monocultures; although no significant difference was observed between either condition, a tendency to decrease the number of infectious virus was observed in the culture as shown in **Figure 6D**, suggesting that moDCs promote fibroblasts protection against DENV-2 infection.

**Table 1:** Reduction of the infection between the mono-cultures and co-cultures.

Donor	% maximum infection in fibroblasts 24hpi	% infection in co-culture F/DC 24hpi	% reduction of infection 24hpi	% maximum infection in fibroblasts 48hpi	% infection in co-culture F/DC 48hpi	% reduction of infection 48hpi
D27	36.28	12.55	65-54	18.28	12.66	30.74
D28	21.1	13.7	35.37	23.4	15.9	32.05
D34	60.16	42.53	25.49	37.4	22.9	38.68
D35	12.23	8.9	27.22	13.83	9.53	31.09
D37	6.17	4.09	66.28	6.29	6.29	0

### The Type I IFN Response Is Upregulated in Fibroblasts-moDCs Cocultures

**Figure 7:**
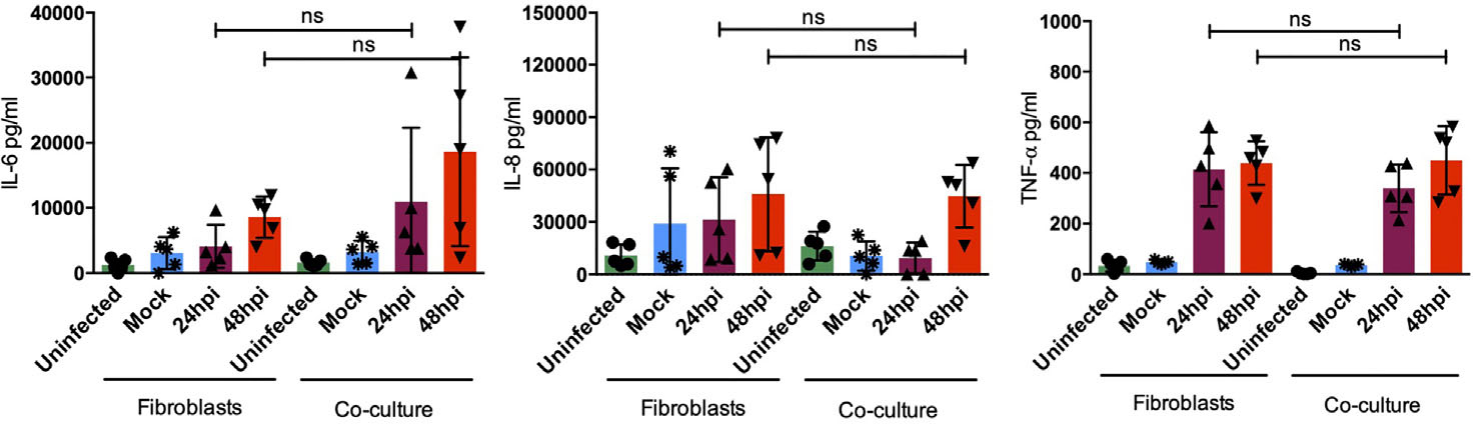
The pro-inflammatory environment is not affected by dendritic cells. Pro-inflammatory cytokine evaluation in HDF and HDF:moDC cocultures after the specified infection times. Error bars indicate the standard deviations for the three independent experiments of each donor; n = 5. ns, no significant.

**Figure 8:**
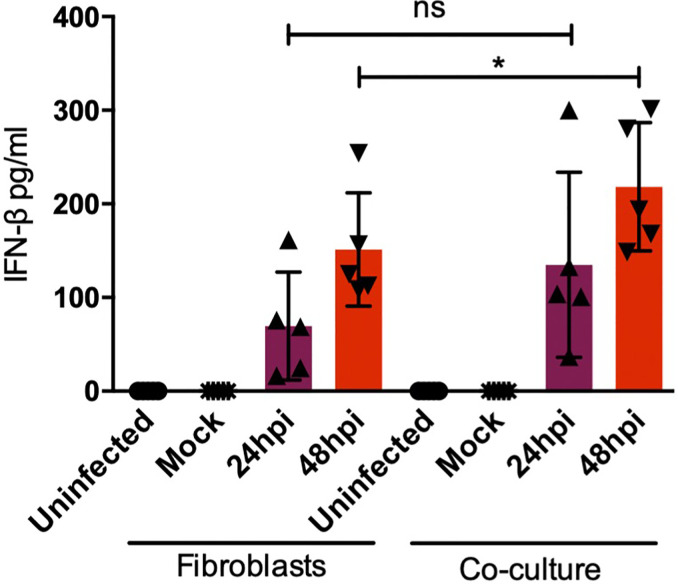
nterferon β response in cocultures is enhanced by dendritic cells. Interferon β secretion in the monocultures and cocultures was assessed using ELISA at 24 and 48 hours after DENV-2 infection. Data are representative of at least three independent experiments for each donor; n = 3. *P < 0.05.

After sensing the external stimulus from a pathogen, sentinel cells in the skin, namely DCs or fibroblasts, acquire the ability to produce cytokines, chemokines, antimicrobial peptides, and type I interferons as part of the immediate innate immune response. All of these molecules modify the local microenvironment and will affect surrounding cells. Among the most abundant cytokines produced in the skin, IL-8, a strong neutrophil chemoattractant, and IL-6, a strong inductor of keratinocyte proliferation and DC activation, can be found. Both IL-6 and IL-8 are highly produced by fibroblasts in the steady state and upon activation, by sensing of pathogens or during inflammation. However, cytokines such as TNF-α are only induced upon activation and poorly produced in the steady state (6, 19). To determine the molecules induced by dermal fibroblasts and dendritic cells in the skin during DENV-2 infection, proinflammatory cytokines were assessed using ELISA in the monocultures and the cocultures. **Figure 7** shows the quantification of proinflammatory cytokines in both monoculture and coculture conditions; although no significant difference was observed among either group, four of the donors showed an increased secretion of IL-6 after 48 hours of infection in the cocultures compared to the monocultures. Additionally, it was interesting to see how each donor cell behaved in different manners, which supports the idea that each patient has its own response. In contrast, IL-8 and TNF-α, respectively, showed no significant difference in either condition. Interestingly, both showed a tendency to decrease after 24 and 48 hours of infection. As for the antiviral microenvironment, IFN-β was also analyzed (**Figure 8**). This cytokine was enhanced at both 24 and 48 hours of infection in the coculture, suggesting that even though the collaboration between DCs and fibroblasts inhibits inflammation, the antiviral state is promoted.

## Discussion

DENV-2 infection is initiated after inoculation of the virus into the skin during the feeding process of an infected mosquito. Many cells in the skin have been shown to support DENV-2 replication, from the widely studied DCs to non-hematopoietic cells, such as fibroblasts and keratinocytes (5, 7). The ability of immune and non-immune skin cells to respond to infection has granted the skin the role of an immune organ, since immunological processes occur here (23). The capacity of the skin to respond to external and internal stimulus is mediated by the response of each cell type with its own signature of secreted molecules, which together form a microenvironment that affects resident cells and the ones recruited into the site of the stimulus. This suggests that tissue resident cells establish a dynamic communication to prevent pathogen dissemination, clearing, and tissue repair (24, 25). To understand a vector-transmitted disease, such as dengue, the skin as its portal of entry must be taken into account.

The aim of our study was to characterize the interplay between dermal fibroblasts and DCs during dengue virus infection to understand how the skin microenvironment is modified by these cell types and how they function during early DENV-2 infection. Data from our group and others have established that the crosstalk between dermal fibroblasts and endothelial cells promotes viral control associated with an enhancement of type I interferon and endothelial activation (19). It has also been observed in human skin explants infected with DENV-2 that the IL-1β produced in the epidermis promotes the infection, recruitment, and migration of myeloid cells (20). These two studies show duality in the response of dendritic cells, where the mediators produced by DCs can both enhance a protective immune response or promote infection. Our data, combined with the previous literature, indicate that the outcome of a productive DENV-2 infection depends on the additive effects of the mediators produced by all cell types present, including their capacity to sense the virus and respond to the microenvironment. We established a study model using autologous dermal fibroblasts and monocyte derived DCs to understand the crosstalk between these two cell types and to determine how their interplay affects the outcome of dengue virus infection.

As a first approach, considering that DCs represent the main link between the innate and adaptive immune response and the close proximity that these cells maintain with fibroblasts in the dermis (17), the activation phenotype of DCs was evaluated in response to conditioned media from DENV-2 infected fibroblasts (FCM). Our results demonstrated that both uninfected and infected fibroblasts are capable of upregulating the surface expression of moDC maturation markers. It seems that the effect of fibroblasts on DCs depends on the type of stimulus and their anatomical location, as previous reports have demonstrated that dermal fibroblasts and dermal dendritic cells come in direct contact in atopic skin, and trans-well and coculture of fibroblasts with moDCs show that fibroblasts promote the maturation of moDC (17). However, in other inflammatory models such as pulmonary and liver fibrosis, fibroblasts seem to suppress DCs maturation (26, 27). After a DC has internalized an antigen, it upregulates chemokine receptors such as CCR7 to migrate out to secondary lymph nodes, present antigen to T cells, and initiate the adaptive immune response (25). During dengue virus infection, the T cell numbers increase during the acute phase, and it has been observed that their numbers severely decrease in patients who undergo critical illness, while this phenomenon does not occur in subjects with mild disease (28). The maturation phenotype of DCs in dengue infected individuals can be correlated with the low T cell numbers in patients with severe disease. It has been observed that myeloid DCs show a lower expression of CD40 and CD86, which would make them inefficient at inducing T cell proliferation (29). Next, moDC maturation occurred in response to DENV-2-FCM, and the permissiveness of these cells to DENV-2 infection showed that DENV-2 infected fibroblasts protected moDC and production of type I interferon was enhanced.

To the best of our knowledge, this is the first time that the effect of stromal cells over DCs has been reported in the context of a viral infection; however, it has been well described that stromal cells can enhance DC function as shown by Saalbach et al., where MMP-9 secretion is induced by IL-6 derived from activated fibroblasts. It has also been observed that endothelial cells can induce the maturation and secretion of IL-12 in dendritic cells (30, 31). It is also known that inflammation will change the network of immune cells: DENV-2 and herpes simplex virus-1 (HSV-1) cause Langerhans cells and dermal dendritic cells to migrate to lymph nodes (20, 32), monocyte recruitment serves to replenish LCs (Langerhans cells arise from monocytes *in vivo*), and monocytes also differentiate into moDC as observed in contact hypersensitivity reactions (33). Therefore, to evaluate the effect of DENV-2 infected fibroblasts in the capacity of inflammatory CD14^+^ monocytes to differentiate into dendritic cells, monocytes were stimulated with GM-CSF and IL-4, plus the conditioned media. All FCM prevented the upregulation of surface CD1a, one of the main moDC markers, and the effect was more severe by mediators secreted by DENV-2 infected fibroblasts. Others have observed that *in vitro* differentiation of moDC renders a CD1a^+^ and a CD1a^-^ population, which have different functions. It has been reported that CD1a^-^ moDCs have lower functionality, since they produce lower amounts of IL-12p70 and induce less T cell proliferation, compared to their CD1a^+^ counterparts, but their phagocytic capacity and secretion of proinflammatory cytokines is not affected (34, 35). Interestingly, type I interferons have been observed to suppress moDC differentiation *in vitro*, even in the presence of GM-CSF and IL-4; low CD1a expression, a lack of dendrites, lower ability to mature, and defective T cells stimulation were observed in type I interferon treated moDC (36).

Under the microscope, high amounts of debris were observed in some donor cultures, and survival did not seem to be affected in other samples; however, in all moDCs cultures independent of the source, lower expression levels of CD1a and also lower percentage of CD1a^+^ cells was observed (data not shown). Donor cultures that did not show lower survival did show the regular dendritic cell-like morphology and were also non-adherent after 6 days of culture, suggesting that this CD1a^-/low^ population forms part of a different moDC type with unknown characteristics. A similar phenomenon was recently observed, where human extra villous trophoblasts cocultured with monocytes inhibited moDC differentiation in a MCP-1 and M-CSF dependent mechanism; this was proposed as a mechanism of immunosuppression that is required during the first stages of pregnancy (37). It has also been observed that monocytes cocultured with gingival fibroblasts, or treated with fibroblast conditioned media, can suppress DC differentiation and also affect T cell proliferation in an IL-6 dependent manner (38).

In the last part of our study, we aimed to understand how DENV-2 infection of dermal fibroblasts can be affected by DCs. After co-culturing HDF with moDCs, the cultures were infected with DENV-2 and the infection rate was evaluated in the cocultures comparing with fibroblast monocultures. Our results showed that moDC promote the control of DENV-2 infection on fibroblasts, as coculture experiments showed a decrease in the percent of infected cells compared to DENV-2 infected fibroblasts in monoculture. It is well known that fibroblasts are strong producers of IFN-β; however, since infection increased after 48 hours, we believe that DENV-2 overcomes the antiviral mechanisms of this cell type and therefore are not capable of completely limiting DENV-2 infection by themselves. Cocultures showed that infection is controlled by enhancing the antiviral response on fibroblasts, by producing factors that promote their protection; according to a similar study reported previously, cocultures of mice DC with mouse cytomegalovirus (MCMV) infected fibroblasts or endothelial cells showed that infection decreases when DCs are added to the culture by promoting the production of IFN-β (39). Although IFNß produced by DENV infected HDF might be playing a key role in the activation of DC, we cannot attribute the different levels of activation of DC solely to this cytokine. Other soluble molecules such as antimicrobial peptides might synergize or antagonize the activation of DC, and more studies are needed to characterize the signature of the soluble mediators and physical signals in the skin microenvironment that can induce protective adaptive immunity.

On other hand flow, cytometry data reveal all cells in the culture, and we cannot rule out the possibility that since DCs are more permissive to DENV infection, the DCs could get infected and die *via* apoptosis, which would reduce the number of infected cells. The physical interactions between HDF/DCs *via* surface molecules such as Thy-1/Mac-1 (CD11a-CD18) or ICAM-1/LFA-1 (CD11b-CD18) induce activation of both HDF and DCs, promoting the secretion of type I interferons previous to the infection.

Our observations lead to the conclusion that the soluble factors released by DENV infected fibroblasts and DCs modify the activation and maturation phenotype of each other, promoting the control of the infection and affecting the differentiation of incoming cells and the developing adaptive immune response. Thus, fibroblasts are more important than originally thought; this has been observed in chronical diseases and during infections, such as those that start in the skin. Furthermore, the final outcome of the infection depends on the sum of the host’s multiple factors, such as the immune status, the previous immunity to heterologous dengue serotypes or other flaviviruses, risk HLA alleles associated with severe disease, among others.

Furthermore, the information provided in our work helps to understand the initial events during DENV infection and might contribute to the understanding of why some infected patients develop only a mild disease, while others are at higher risk to develop severe disease.

## Data Availability Statement

The datasets generated for this study are available on request to the corresponding author.

## Ethics Statement

The studies involving human participants were reviewed and approved by Comité de enseñanza, capacitación Investigación y Etica Del Hospital general de Ticoman Secretaria de Salud Ciudad de Mexico. The patients/participants provided their written informed consent to participate in this study.

## Author Contributions

Conceived and design of the experiments: AM-G, MS-T, LS-A, JB-A, and LC-B. Performed the experiments: AM-G, JG-C, GV-C, EM-J, FJ-D, and GS. Analyzed the data: AM-G, JG-C, EM-J, MS-T, LS-A, LF-R, and LC-B. Wrote the paper: AM-G, JB-A, and LC-B. All authors contributed to the article and approved the submitted version.

## Funding 

This work was supported by CINVESTAV LC-B and partiallly supported by CONACyT, grant PN2029 JB-A.

## Conflict of Interest

The authors declare that the research was conducted in the absence of any commercial or financial relationships that could be construed as a potential conflict of interest.
